# Rhabdomyolysis Due to Salmonellosis: A Case Report of a Rare Presentation

**DOI:** 10.7759/cureus.19781

**Published:** 2021-11-21

**Authors:** Ana Mestre, Guilherme Cunha, Natália Teixeira, Cátia Correia, Yahia Abuowda

**Affiliations:** 1 Internal Medicine, Hospital Distrital de Santarém, Santarém, PRT

**Keywords:** fluid replacement, acute kidney injury, gastroenteritis, salmonella infection, rhabdomyolysis

## Abstract

*Salmonella* infection has been commonly associated with gastrointestinal symptoms, such as diarrhea, abdominal pain, and nausea. However, in some cases, patients can develop rare and life-threatening complications such as rhabdomyolysis.

Here, we report a case of gastroenteritis due to *Salmonella enteritidis* infection complicated by rhabdomyolysis. The patient was successfully treated with fluids and antibiotics.

Despite rare, the association of *Salmonella* infection and rhabdomyolysis may occur. Prompt diagnosis and treatment with aggressive fluid replacement and antibiotics are paramount to prevent acute kidney injury.

## Introduction

*Salmonella* remains one of the most common causes of gastrointestinal infection in the world and can cause two types of disease: gastroenteritis, associated with non-typhoidal salmonella, and enteric fever, caused by *Salmonella* typhi or paratyphi [1]. Although infection by *Salmonella* has been commonly associated with diarrhea, rare extraintestinal complications such as pancreatitis, myocarditis, and multi-organ failure have been reported [2-4].

Rhabdomyolysis is a rare and potentially severe complication of *Salmonella* infections [5]. Its mechanism is not fully understood; however, it is thought that dehydration and electrolyte disturbances, as well as the direct effects of the bacterial toxin on cell metabolism, can lead to muscle damage [6].

Here, we report a case of rhabdomyolysis due to *Salmonella* infection.

## Case presentation

A previously healthy 29-year-old Caucasian female presented to the emergency department with diffuse myalgia, abdominal discomfort, and watery diarrhea for three days. There was no history of fever, intense physical exercise, drug, or alcohol consumption. She denied any recent travel.

On physical examination, she was dehydrated. On admission, her vital signs were pulse rate 90 beats/min, blood pressure 110/62 mmHg, respiratory rate 18 breaths/min, and temperature 36.4ºC. General abdominal tenderness without hepatosplenomegaly was noted on palpation. Rash was absent, and no muscular weakness was noted.

Laboratory testing showed mild leukocytosis, with predominant neutrophilia and normal platelets (PLT). Biochemistry analysis yielded markedly elevated creatine kinase (CK) (Table 1). High serum levels of lactate dehydrogenase (LDH), aspartate aminotransferase (AST), and alanine aminotransferase (ALT) were also present. Further laboratory results namely renal function, electrolytes, alkaline phosphatase, gamma-glutamyl transferase, amylase, and total bilirubin were within the reference range. C-reactive protein (CRP) level was 2.68 mg/dL. Urinalysis showed muddy brown urine and was positive for myoglobin. HIV was negative. The electrocardiogram was normal. An abdominal ultrasound showed normal liver, spleen, and kidney. Due to the marked elevation of the muscle enzymes, it was decided to be admitted to the Internal Medicine ward for further study and monitoring.

**Table 1:** Evolution of laboratory testing values during hospitalization from admission to discharge Hb - hemoglobin, WBC - white blood cell count, PLT - platelets, ALT - alanine aminotransferase, AST - aspartate aminotransferase, CK - creatine kinase, and LDH - lactate dehydrogenase.

Day of admission	1	2	3	4	5	6
Hb (g/dL)	17.4	15.2	14.2	14.1	14.2	13.8
WBC (10^9^/L)	10.1	8.7	8.2	4.2	4.5	4.2
PLT (10^9^/L)	225	228	171	201	162	180
Urea (mg/dL)	30.0	24.3	13.2	15.0	11.2	11.7
Creatinine (mg/dL)	0.9	0.8	0.8	0.7	0.8	0.8
Serum Sodium (mmol/L)	141	140	138	139	138	138
Serum Potassium (mmol/L)	4.0	4.0	3.9	3.8	4.0	3.8
ALT (U/L)	342	272	248	172	90	41
AST (U/L)	86	69	73	61	58	52
CK (U/L)	20741	13321	5267	1536	658	223
LDH (U/L)	851	521	312	264	193	177

Initially, intravenous hydration was given with good clinical response and normalization of the laboratory parameters. On the third day of hospitalization, *Salmonella enteritidis* was isolated from stool cultures and was prescribed oral ciprofloxacin. Blood cultures were sterile.

The patient was discharged six days after admission with a prescription for oral ciprofloxacin to complete a seven-day course of treatment. She had no symptoms, and enzyme levels had dropped significantly. After two weeks, the patient had a follow-up clinic appointment, she remained asymptomatic, and the laboratory tests were completely normal.

## Discussion

Rhabdomyolysis is characterized by the injury and necrosis of skeletal muscle tissue with the consequent release of intracellular content such as myoglobin, CK, aminotransferases, and LDH into the bloodstream [7]. Typical clinical manifestations are pain and muscle weakness, and dark urine may be present. However, the clinical presentation can vary from an asymptomatic increase in serum levels of these enzymes to potential life-threatening acute kidney injury (AKI) [8]. A systematic review showed that most studies define rhabdomyolysis by a specific CK cutoff value, the majority of which is a CK level > 1,000 U/L or at least five times the upper limit of normal [9]. Our patient presented myalgia and markedly elevation of CK level. Despite her serum creatinine being normal, it is important to notice that increasing CK activity in rhabdomyolysis has been associated with a higher incidence of AKI [10].

Multiple etiologies of rhabdomyolysis have been described. It can be due to common and non-infectious causes such as trauma, extreme exertion, ischemic disorders, drugs and toxins, and more rarely genetic causes (enzyme deficiencies and myopathies); but has also been reported in association with viral (e.g., influenza, Epstein-Barr virus, Coxsackievirus, and HIV) and bacterial infections (e.g., *Streptococcus pneumoniae*, *Legionella*, *Staphylococcus,* and *Escherichia coli*) [1,8]. Although rare, rhabdomyolysis secondary to *Salmonella* infection has also been described in the literature [2,6,11]. In the present case, the initial clinical presentation was highly suggestive of gastroenteritis, and the isolation of *Salmonella* from stool cultures confirmed the diagnosis of salmonellosis. The other causes of rhabdomyolysis were ruled out, including trauma, extreme exercise, medications, illicit drugs, auto-immune disease, and other infections. Genetic causes were considered unlikely in this case since these conditions are typically associated with recurrent episodes and present in childhood.

The underlying mechanism of *Salmonella*-induced rhabdomyolysis remains unclear. It was suggested that tissue hypoxia, toxin release, and direct bacterial invasion of muscle could be responsible for muscle cell injury [6]. The progression to AKI due to rhabdomyolysis seems to result from several mechanisms such as direct and ischemic tubule injury, formation of toxic free radicals, renal vasoconstriction, and tubular obstruction caused by myoglobin [8].

Rhabdomyolysis treatment includes two components: correction of the underlying cause of the muscle injury and prevention of AKI progression and associated metabolic abnormalities. Early and adequate hydration is the foundation for preventing and treating rhabdomyolysis-induced AKI. Intravenous fluid administration dilutes nephrotoxins and increases glomerular filtration and urine flow, preventing the accumulation of myoglobin and limiting the formation of intratubular casts [12]. The importance of fluid replacement is established. However, there are no standardized guidelines that define the type of fluid or the adequate volume needed. A target of 6 to 12 L within 24 h is a reasonable goal, and fluid administration should be maintained until the resolution of rhabdomyolysis or until plasma CK level is under 5000 U/L [8,12]. The volume status and urine output should be carefully monitored, and fluid rates adjusted as necessary to avoid volume overload. There is small evidence to support urine alkalinization with bicarbonate administration or the use of mannitol and loop diuretics [12]. When AKI is established and fails to reverse despite fluid replacement, dialysis may be necessary to correct severe metabolic abnormalities and manage volume overload. In the current case, the underlying cause of rhabdomyolysis was treated with adequate antibiotics and fluid replacement, which led to a resolution of symptoms, and CK level returned to baseline.

## Conclusions

*Salmonella* infections usually present with mild gastrointestinal symptoms; however extraintestinal manifestations, such as rhabdomyolysis, can represent a rare and deadly complication. Skeletal muscle manifestations may range from mild myalgias to severe muscle pain, making rhabdomyolysis often underdiagnosed.

Early recognition and treatment with aggressive fluid replacement and antibiotics are essential to the successful management of *Salmonella*-induced rhabdomyolysis.
